# Oxytrodiflavanone A and Oxytrochalcoflavanones A,B: New Biflavonoids from *Oxytropis chiliophylla*

**DOI:** 10.3390/molecules24081468

**Published:** 2019-04-13

**Authors:** Yang Liu, Norbo Kelsang, Jianghai Lu, Yingtao Zhang, Hong Liang, Pengfei Tu, Dexin Kong, Qingying Zhang

**Affiliations:** 1 State Key Laboratory of Natural and Biomimetic Drugs and Department of Natural Medicines, School of Pharmaceutical Sciences, Peking University Health Science Center, Beijing 100191, China; 1311210122@bjmu.edu.cn (Y.L.); kelsang815@163.com (N.K.); zytao1988@163.com (Y.Z.); lianghong@bjmu.edu.cn (H.L.); pengfeitu@bjmu.edu.cn (P.T.); 2 National Anti-Doping Laboratory, China Anti-Doping Agency, Beijing 100029, China; lujianghai@chinada.cn; 3 Tianjin Key Laboratory on Technologies Enabling Development of Clinical Therapeutics and Diagnostics, School of Pharmacy, Tianjin Medical University, Tianjin 300070, China; 4 Research Center, School of Medicine, Tianjin Tianshi College, Tianyuan University, Tianjin 301700, China

**Keywords:** *Oxytropis chiliophylla*, *Oxytropis*, biflavonoids

## Abstract

Three previously undescribed biflavonoids, oxytrodiflavanone A (**1**), and oxytrochalcoflavanones A,B (**2**,**3**), were isolated from the aerial part of *Oxytropis chiliophylla*, together with their putative biosynthetic monomers, i.e., (2*S*)-5,7-dihydroxyflavanone (**4**), (2*S*)-7-hydroxyflavanone (**5**), and 2′,4′-dihydroxychalcone (**6**). The structures of these compounds were elucidated by a combination analysis of spectroscopic data. The cytotoxic activities of all the isolated compounds against PC-3 human prostate cancer cell line are also presented.

## 1. Introduction

**Figure 1:**
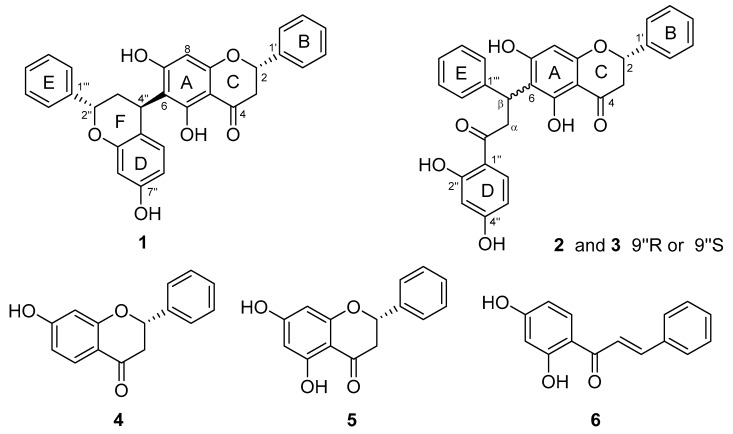
Structures of compounds **1**–**6**.

*Oxytropis chiliophylla* Royle ex Benth (Leguminosae), along with *Oxytropis falcata* Bunge, is recorded as the official botanical origin of Tibetan medicine “Er-Da-Xia” in Chinese Pharmacopoeia, which is regarded as the “King of Herbs” and has been widely used in Tibetan medicines for the treatment of inflammation, pyreticsis, and bleeding for thousands of years [1,2]. Mainly found at the altitudes of 2800–5200 meters in Tibet and Xinjiang Urgur Autonomous Regions in China [3], *O. chiliophylla* was reported to contain a large number of flavonoids as the major bioactive constituents [4,5,6,7]. Of these flavonoids, structurally diverse cytotoxic chalcone dimers, isolated from the aerial parts of *O. chiliophylla* by our group, were of great significance in interpreting the hypothesized ecological functions of the chemicals [7]. In an ongoing investigation on the structurally unique and bioactive constituents from *O. chiliophylla*, we particularly focused on the isolation and identification of other biflavonoids guided by LC-QTOF/MS (Liquid Chromatograph-Quadrupole Time-of-Flight/Mass Spectrometer). As a result, three previously undescribed biflavonoids, oxytrodiflavanone A (**1**) and oxytrochalcoflavanones A,B (**2**,**3**) (Figure 1), were obtained from the aerial parts of *O. chiliophylla*. In addition, their putative biosynthetic monomers, i.e., (2*S*)-5,7-dihydroxyflavanone (**4**), (2*S*)-7-hydroxyflavanone (**5**), and 2′,4′-dihydroxychalcone (**6**), were also obtained from the herb. The cytotoxic activities of all isolates were evaluated against the PC-3 human prostate cancer cell line.

## 2. Results and Discussion

**Figure 2:**
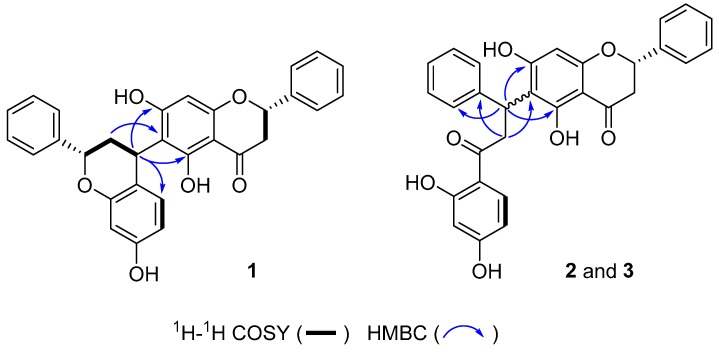
Key ^1^H-^1^H COSY (^1^H-^1^H Correlation Spectroscopy) and HMBC correlations of compounds **1**–**3**.

**Figure 3:**
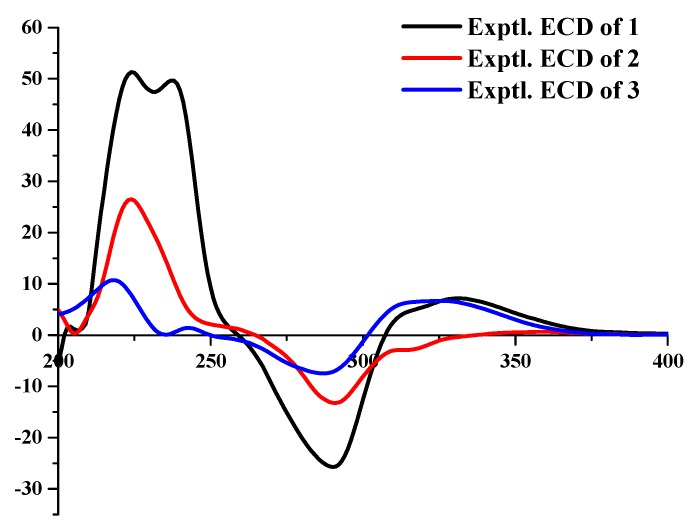
ECD spectra of compounds **1**–**3**.

**Scheme 1:**
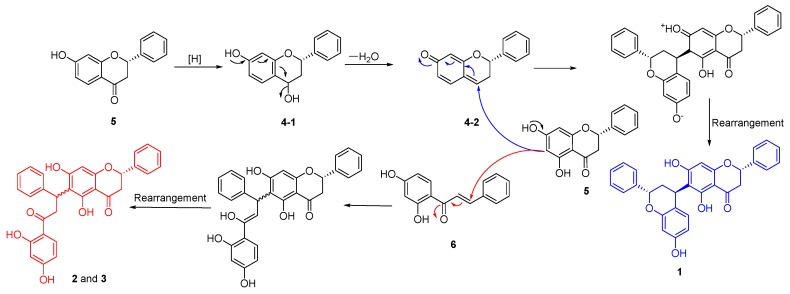
Plausible biosynthetic pathway of compounds **1**–**3**.

Oxytrodiflavanone A (**1**), obtained as yellow amorphous powders, had a molecular formula of C_30_H_24_O_6_ based on the deprotonated molecular ion at *m*/*z* 479.1499 [M − H]ˉ (calcd for C_30_H_23_O_6_, 479.1500) in its negative HR-ESIMS (High resolution-Electric Spray Ion Mass Spectrometer) spectrum and NMR (Nuclear Magnetic Resonance) data. The UV (Ultraviolet) spectrum showed maximum absorption bands at 235 and 283 nm. The ^1^H- and ^13^C-NMR data of **1** (Table 1) exhibited characteristic signals for a biflavonoid consisting of one 5,6,7-trisubstituted flavanone residue and one 4,7-disubstituted flavane moiety, evidenced by a set of ABX-spin aromatic signals at *δ*_H_ 6.98 (1H, d, *J* = 8.4 Hz), 6.55 (1H, d, *J* = 2.1 Hz), and 6.45 (1H, dd, *J* = 8.4, 2.1 Hz), overlapped signals for two monosubstituted benzene rings at *δ*_H_ 7.35–7.46 (10H, m), an aromatic singlet at *δ*_H_ 6.00 (1H, s), a CH–CH_2_ spin system at *δ*_H_ 5.42 (1H, dd, *J* = 13.1, 2.5 Hz), 3.09 (1H, dd, *J* = 17.2, 13.1 Hz), and 2.84 (1H, dd, *J* = 17.2, 2.5 Hz), a CH–CH_2_–CH spin system at *δ*_H_ 5.30 (1H, t, *J* = 5.0 Hz), 4.52 (1H, t, *J* = 5.9 Hz), and 2.40 (2H, t, *J* = 5.9 Hz), and the corresponding ^13^C-NMR signals. The 1D- and 2D-NMR spectra allowed the assignment of all signals. Based on the MS and NMR data, three hydroxyl groups were deduced to be present. The characteristic hydrogen-bonded hydroxyl resonance at *δ*_H_ 12.58 (1H, s) indicated the presence of 5-OH, and the other two hydroxyl groups were placed at C-7 and C-7″, respectively, based on the 1D and 2D-NMR data. The HMBC (Heteronuclear Multiple Bond Connectivity) correlations between H-4″ and C-5, C-7, between H-3″ and C-6 (Figure 2) established the C-6 and C-4″ linkage between the two flavonoid units, which was further confirmed by the characteristic fragment ions at *m*/*z* 223.0760 and 255.0664 in the targeted MS/MS data that attributed to the cleavage of bond between C-6 and C-4″. The 2*S* configuration of **1** was determined according to the positive Cotton effect at 320~330 nm and negative effect at 270–290 nm (Figure 3) [8], and the 2″*S* configuration of **1** was tentatively inferred based on the biosynthetic consideration (Scheme 1). The relative configuration of F-ring of **1** was deduced to be the same as that of friesodielsone A and desmosflavan A [9,10] according to the coupling constants for H-2″, H-3″, and H-4″, and further supported by the absence of NOE interaction between H-2″ and H-4″. The absolute 4″*S* configuration was evident from the positive Cotton Effect at 225.0 nm (Figure 3) [11,12,13], and thus allowed the assignment of 2″*S* configuration, which was consistent with the above deduction based on biosynthetic consideration. Unambiguously, the structure of **1** was characterized as shown in Figure 1 and named oxytrodiflavanone A.

Oxytrochalcoflavanones A (**2**) and B (**3**), yellow amorphous powders with the same molecular formula of C_30_H_24_O_7_ based on the negative HR-ESIMS data, were also found to be biflavonoids. The UV, ECD (Electric Circular Dichroism), ^1^H and ^13^C-NMR spectra of **2** and **3** (Table 2) were almost identical, but with the opposite optical rotations ([α]^20^_D_ −68.6 for **2**; [α]^20^_D_ +51.9 for **3**). The main differences between the ^1^H- and ^13^C-NMR data of **2**, **3** and **1** were the presence of signals for a 2′,4′-dihydroxy dihydrochalcone moiety in **2** and **3** instead of those for the 7-hydroxy flavane moiety in **1**. Identical HMBC correlations between H-β and C-5, C-7, and between H-α and C-6 in **2** and **3** defined the same connectivity of C-β and C-6 in **2** and **3**, which was supported by the characteristic fragment ions at *m*/*z* 239.0350 and 255.0667 in targeted MS/MS spectra attributed to the bond cleavage of C-6 and C-β. Thus, the same planar structures of **2** and **3** were defined as shown in Figure 1. Accordingly, the 2*S* configurations of **2** and **3** were assigned based on the Cotton effect (Figure 3) [8]. The absolute configuration of C-β could not be determined by comparing the experimental and calculated ECD spectra due to the close similarity in their ECD curves (Figure 3) and attempts to get single crystals of **2** and **3** also failed. Consequently, compounds **2** and **3** were characterized as the C-β epimers, but with undefined absolute configuration of C-β. 

Compounds **1**–**3** were obtained under the guidance of LC-QTOF/MS in negative ionization mode by extracting the corresponding deprotonated molecular ion [M − H]^−^. Other potential biflavonoids with different skeletons and substituents were also analyzed but not detected, which might be due to the much low abundance in the herb. Biosynthetically, compounds **1**–**3** were proposed to be derived from compound **4** via reduction of the C-4 carbonyl group, formation of 4-2 by dehydration, and then coupled with **5** and **6** respectively (Scheme 1). This biosynthetic hypothesis is supported by the isolation of compounds **4**–**6** from this herb.

All the isolated compounds were evaluated for cytotoxic activities against PC-3 human prostate cancer cell line. Compounds **1** and **3** exhibited potent cytotoxicity with IC_50_ values of 6.64 μM and 2.98 μM (Figure S16, Table S1), whereas compound **2** showed moderate cytotoxicity whose IC_50_ value was not calculated.

## 3. Experimental Section

### 3.1. General Experimental Procedures

Optical rotations were measured on an Autopol III Rudolph Automatic polarimeter. ECD data were acquired on a Jasco 810 CD spectrophotometer. UV spectra were recorded using a Shimadzu SPD-M10A spectrophotometer. NMR spectra were recorded on Bruker Plus-400 or Advance-500 NMR spectrometer using CDCl_3_ as solvents. Chemical shift values were given in δ (ppm) using the solvent peak signals as references, and coupling constants were reported in Hz. HR-ESIMS experiments were conducted on an Agilent 6538 QTOF mass spectrometer. Column chromatography was performed on silica gel (100–200 mesh or 200–300 mesh, Qingdao Marine Chemical Co. Ltd., Qingdao, China), ODS-A (50 μm, YMC Co. Ltd, Tokyo, Japan), or Sephadex LH-20 (GE Healthcare Bio-Sciences AB, Uppsala, Sweden). Preparative HPLC was carried out using a GRACE Alltima RP-C18 column (10 × 250 mm, 5 μm) on a JASCO PU-1580 LC instrument with a diode array detector (DAD) or a CHIRALPAK ID (4.6 × 250 mm, 5 μm, Daicel Chemical Ind., Ltd., Tokyo, Japan) on a Jasco HPLC system coupled with a single wavelength detector. TLC analyses were carried out on pre-coated silica gel GF254 plates (Qingdao Marine Chemical Co. Ltd., Qingdao, China). Reagents used in HPLC and LC-MS analyses were of HPLC-grade purchased from J. T. Baker. Deionized water was purified by a Milli-Q system (Bedford, MA, USA). All other solvents used for isolation and analysis were of analytical grade purchased from Beijing Fine Chemicals (Beijing, China), and used without further purification. The CDCl_3_ used in the NMR studies were purchased from Cambridge Isotope Laboratories, Inc. (Andover, MA, USA).

### 3.2. Plant Material

The whole plants of *O. chiliophylla* were collected from Nagarzê County of Tibet Autonomous Region, People’s Republic of China. The plants were immediately air-dried and stored in a warehouse at room temperature out of direct sunlight. The authenticity of the herb was confirmed by Associate Prof. Yingtao Zhang, Department of Natural Medicines, School of Pharmaceutical Sciences, Peking University Health Science Center, and a voucher specimen (No. 20120901) was deposited in the Herbarium of the Department of Natural Medicines, School of Pharmaceutical Sciences, Peking University Health Science Center.

### 3.3. Extraction and Isolation

The isolation of biflavonoids (**1**–**3**) was guided by LC-QTOF/MS. Each fraction was analyzed by LC-QTOF/MS through extraction of the quasi-molecular ions and then purified by repeated chromatography. The air-dried aerial parts of *O. chiliophylla* (10.0 kg) were pulverized and extracted with 95% EtOH and 50% EtOH successively to afford two crude extracts. After concentration in vacuum, the 95% EtOH and 50% EtOH extracts were separately suspended in water and partitioned sequentially with petroleum ether (40 L × 3), EtOAc (50 L × 3), and n-BuOH (60 L × 3). The EtOAc fractions were combined (900 g) and subjected to silica gel column chromatography eluted with a gradient system of petroleum ether/EtOAc (from 10:1 to 1:5, *v*/*v*) to afford fractions A–H. Fraction C (15.0 g) was purified by Sephadex LH-20 column chromatography eluted with an isocratic elution of petroleum ether/CH_2_Cl_2_/MeOH (5:5:1, *v*/*v*/*v*) to yield subfractions Ca–Cf. Purification of subfractions Ce–Cf by semi-preparative HPLC eluting with a MeOH/H_2_O system yielded compound **1** (1.2 mg) and the mixture of compounds **2** and **3**. Failed in the separation of **2** and **3** by a conventional RP-C18 column, compounds **2** (0.8 mg) and **3** (0.4 mg) were resolved by chiral-phase HPLC using a CHIRALPAK ID column (4.6 × 250 mm). Fraction G (5.0 g) was subjected to repeated Sephadex LH-20 column chromatography eluted with an isocratic elution of petroleum ether/CH_2_Cl_2_/MeOH (5:5:1, *v*/*v*/*v*) to give compounds **4** (100.3 mg), **5** (302.5 mg), and **6** (200.1 mg).

Oxytrodiflavanone A (**1**): yellow amorphous powders; [α]^20^_D_ +70.0 (c 0.1, MeOH); UV (MeOH) 235, 283 nm; (−)-HR-ESIMS *m*/*z* 479.1499 [M − H]^−^ (calcd for C_30_H_23_O_6_, 479.1500); ^1^H- and ^13^C-NMR data see Table 1.

Oxytrochalcoflavanone A (**2**): yellow, amorphous powders; [α]^20^_D_ −68.6 (c 0.1, MeOH); UV (MeOH) 235, 283 nm; (−)-HR-ESIMS *m*/*z* 495.1453 [M − H]^−^ (calcd for C_30_H_23_O_7_, 495.1449); ^1^H- and ^13^C-NMR data see Table 2.

Oxytrochalcoflavanone B (3): yellow amorphous powders; [α]^20^_D_ +51.9 (c 0.1, MeOH); UV (MeOH) 235, 283 nm; (−)-HR-ESIMS *m*/*z* 495.1453 [M − H]^−^ (calcd for C_30_H_23_O_7_, 495.1449); ^1^H- and ^13^C-NMR data see Table 2.

### 3.4. Cytotoxicity Assays against PC–3 Human Prostate Cancer Cells

Human prostate cancer cell line PC-3 was obtained from the American Type Culture Collection (Manassas, VA, USA) and grown according to the recommended guidelines. Cells were cultured in RPMI 1640 medium (HyClone, Cramlington, UK) supplemented with 10% (*v*/*v*) foetal bovine serum (Biological Industries, Kibbutz Beit-Haemek, Israel), 10 μg/mL streptomycin and 100 U/mL penicillin at 37 °C in a humidified atmosphere containing 5% CO_2_. Cell viability was determined using MTT assay [14]. Briefly, following treatment of PC3 cells with various concentrations of the tested drugs for 48 h in 96 well plates, MTT was added to each well at a final concentration of 0.5 mg/mL. After incubated for 4 h at 37 °C, the culture medium was removed, and the purple formazan crystals were dissolved in 150 μL of DMSO. The absorbance of the resulting solution was measured at 490 nm by a microplate reader (iMark, Bio-Rad, Hercules, CA, USA). Cell viability (%) and cell growth inhibition (%) were calculated using the following formulas:

Cell viability (%) = 100 × (absorbance of a given sample – absorbance of blank well)/(absorbance of control well – absorbance of blank well), Cell growth inhibition (%) = 100% – Cell viability (%), where the blank well contained medium but no cells and the control well-contained cells but no drug. The IC_50_ values were calculated by fitting the data points to a logistic curve using GraphPad Prism 5 software (GraphPad Software, San Diego, CA, USA). ZSTK474, an antitumor agent in clinical trials, was used as the positive control.

## 4. Conclusions

Three previously undescribed biflavonoids, oxytrodiflavanone A (**1**), and oxytrochalcoflavanones A,B (**2**,**3**), along with their putative biosynthetic monomers (**4**–**6**), were isolated from the aerial parts of *O. chiliophylla* guided by LC-QTOF/MS. Compounds **1** and **3** exhibited potent cytotoxicity against PC-3 human prostate cancer cell line with IC_50_ values of 6.64 μM and 2.98 μM.

## Figures and Tables

**Table 1:** ^1^H-NMR and ^13^C-NMR data for oxytrodiflavanone A (**1**) (*δ* in ppm, *J* in Hz).

No.	^1^H-NMR ^a^	^13^C-NMR	^1^H-NMR ^b^
1	-	-	-
2	5.42 (dd, 13.1, 2.5)	79.17	5.56 (dd, 13.2, 3.0)
3α	3.09 (dd, 17.2, 13.1)	43.69	17.02 (dd, 17.0, 13.2)
3β	2.84 (dd, 17.2, 2.5)		2.89 (17.0, 3.0)
4	-	196.11	-
5	-	162.07	-
6	-	109.96	-
7	-	164.13	-
8	6.00 (s)	96.99	6.44 (s)
9	-	161.47	-
10	-	103.12	-
1′	-	138.57	-
2′/6′	7.43–7.46 ^c^	126.26	7.24–7.42 ^c^
3′/5′	7.43–7.46 ^c^	129.03	7.24–7.42 ^c^
4′	7.43–7.46 ^c^	129.03	7.24–7.42 ^c^
5-OH	12.58 (s)	-	13.19
1″	-	-	-
2″	5.30 (t, 5.0)	75.76	5.99 (dd, 6.0, 3.6)
3″α	2.40 (t, 5.9)	34.94	3.27 (ddd, 13.8, 8.4, 3.6)
3″β	2.40 (t, 5.9)		2.65 (dt, 13.8, 6.0)
4″	4.52 (t, 5.9)	28.25	5.07 (t, 7.2)
5″	6.98 (d, 8.4)	130.73	7.67 (d, 7.8)
6″	6.45 (dd, 8.4, 2.1)	104.61	6.85 (dd, 8.4, 2.4)
7″-OH	-	156.76	11.35
8″	6.55 (d, 2.1)	109.82	7.15 (d, 2.4)
9″	-	156.25	-
10″	-	112.98	-
1‴	-	140.81	-
2‴/6‴	7.35–7.42 ^c^	126.00	7.24–7.42 ^c^
3‴/5‴	7.35–7.42 ^c^	128.69	7.24–7.42 ^c^
4‴	7.35–7.42 ^c^	127.93	7.24–7.42 ^c^

^a 1^H-NMR (500 MHz) and ^13^C-NMR (125 MHz) in CDCl_3_. ^b 1^H-NMR (600 MHz) in Pyridine-*d*_5_. ^c^ Overlapped signals.

**Table 2:** ^1^H-NMR (500 MHz) and ^13^C-NMR (125 MHz) data for oxytrochalcoflavanones A (**2**) and B (**3**) (*δ* in ppm, *J* in Hz, in CDCl_3_).

No.	^1^H-NMR	^13^C-NMR	No.	^1^H-NMR	^13^C-NMR
1	-	-	H-β	5.10 (dd, 9.4, 4.6)	34.51
2	5.35 (dd, 13.2, 2.8)	79.22	H-α ^a^	4.25 (dd, 17.5, 9.4)	40.03
3α	3.09 (dd, 17.1, 13.2)	43.59	H-α ^b^	3.73 (dd, 17.5, 4.6)	
3β	2.84 (dd, 17.1, 2.8)		1″	-	142.36
4	-	196.19	2″/6″	7.37–7.42 ^a^	127.86
5	-	162.23	3″/5″	7.37–7.42 ^a^	128.37
6	-	111.05	4″	7.37–7.42 ^a^	126.46
7	-	163.20	1‴	-	114.07
8	6.05 (s)	96.89	2‴	-	165.34
9	-	161.51	3‴	6.34 (d, 1.0)	103.68
10	-	103.48	4‴	-	163.23
1′	-	138.51	5‴	6.40 (dd, 8.9, 1.0)	108.21
2′/6′	7.37–7.42 ^a^	126.27	6‴	7.83 (d, 8.9)	132.74
3′/5′	7.37–7.42 ^a^	129.01	5-OH	12.46 (s)	
4′	7.37–7.42 ^a^	129.01	2‴-OH	12.40 (s)	

^a^ Overlapped signals.

## Supplementary Materials

1D- and 2D-NMR spectra, targeted MS/MS spectra, and the raw data about cell growth inhibitory activities are available online.
