# Supplementary material for: Oxytrodiflavanone A and Oxytrochalcoflavanones A,B: New Biflavonoids from Oxytropis chiliophylla
Source: Molecules. 2019 Apr 13;24(8):1468. doi: 10.3390/molecules24081468 (PMC6514731; doi:10.3390/molecules24081468)
Supplement: Supplementary file 1 [file molecules-24-01468-s001.pdf]

Supporting Information for

Oxytrodiflavanone A and Oxytrochalcoflavanones A,B: New Biflavonoids from *Oxytropis chiliophylla*

Yang Liu [1], Norbo Kelsang [1], Jianghai Lu [2], Yingtao Zhang [1], Hong Liang [1], Pengfei Tu [1], Dexin Kong [3, 4,] * and Qingying Zhang [1,]*

[1] State Key Laboratory of Natural and Biomimetic Drugs and Department of Natural Medicines, School of Pharmaceutical Sciences, Peking University Health Science Center, Beijing 100191, China;

[2] National Anti-Doping Laboratory, China Anti-Doping Agency, Beijing 100029, China;

[3] Tianjin Key Laboratory on Technologies Enabling Development of Clinical Therapeutics and Diagnostics, School of Pharmacy, Tianjin Medical University, Tianjin 300070, China

[4] Research Center, School of Medicine, Tianjin Tianshi College, Tianyuan University, Tianjin 301700, China

Contents of Supporting Information

Figures

Figure S1. ^{1}H NMR spectrum of oxytrodiflavanone A (**1**) in $CDCl_3$

Figure S2. ^{13}C NMR spectrum of oxytrodiflavanone A (**1**) in $CDCl_3$

Figure S3. ^{1}H-^{1}H COSY spectrum of oxytrodiflavanone A (**1**) in $CDCl_3$

Figure S4. HSQC spectrum of oxytrodiflavanone A (**1**) in $CDCl_3$

Figure S5. HMBC spectrum of oxytrodiflavanone A (**1**) in $CDCl_3$

Figure S6. ^{1}H NMR spectrum of oxytrodiflavanone A (**1**) in Pyridine-d_5

Figure S7. NOESY spectrum of oxytrodiflavanone A (**1**) in Pyridine-d_5

Figure S8. ^{1}H NMR spectrum of oxytrochalcoflavanones A (**2**) and B (**3**) in $CDCl_3$

Figure S9. ^{13}C NMR spectrum of oxytrochalcoflavanones A (**2**) and B (**3**) in $CDCl_3$

Figure S10. ^{1}H-^{1}H COSY spectrum of oxytrochalcoflavanones A (**2**) and B (**3**) in $CDCl_3$

Figure S11. HSQC spectrum of oxytrochalcoflavanones A (**2**) and B (**3**) in $CDCl_3$

Figure S12. HMBC spectrum of oxytrochalcoflavanones A (**2**) and B (**3**) in $CDCl_3$

Figure S13. Targeted MS/MS spectra of oxytrodiflavanone A (**1**), oxytrochalcoflavanones A (**2**) and B (**3**) at CE of 25 eV in negative ion mode

Figure S14. Chiral analysis of oxytrochalcoflavanones A−B (**2-3**)

Figure S15.^{1}H NMR spectra of the epimers of oxytrochalcoflavanones A−B (**2-3**)

Figure S16. Cell growth inhibitory activities of oxytrodiflavanone A (**1**) and oxytrochalcoflavanones B (**3**) on PC3 cells

Table

Table S1. Raw data of cell growth inhibitory activities of oxytrodiflavanone A (**1**) and oxytrochalcoflavanones B (**3**)

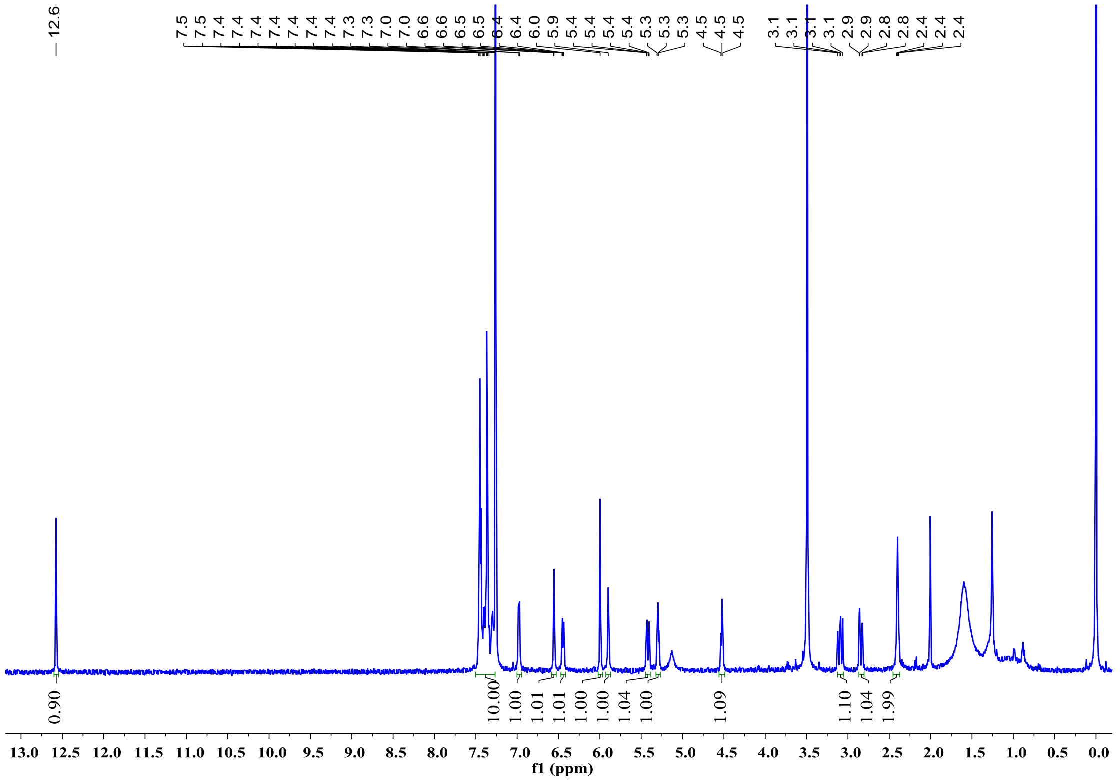

Figure S1. ^{1}H NMR spectrum of oxytrodiflavanone A (**1**) in $CDCl_3$

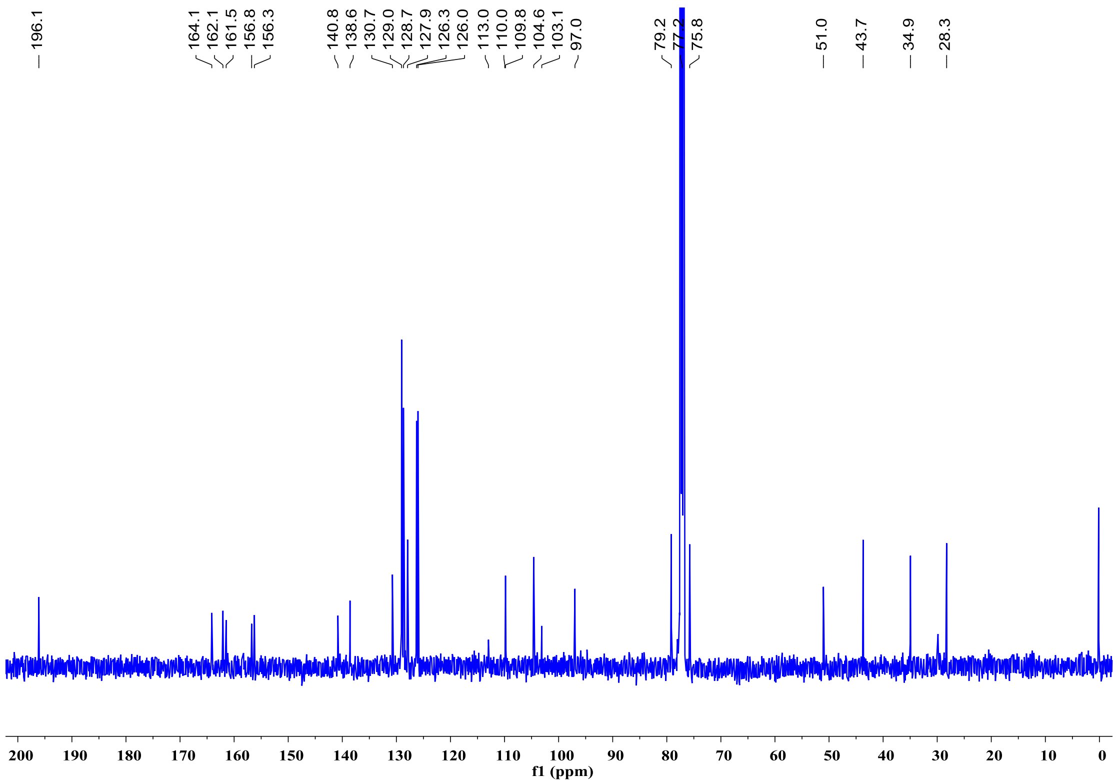

Figure S2. ^{13}C NMR spectrum of oxytrodiflavanone A (**1**) in $CDCl_3$

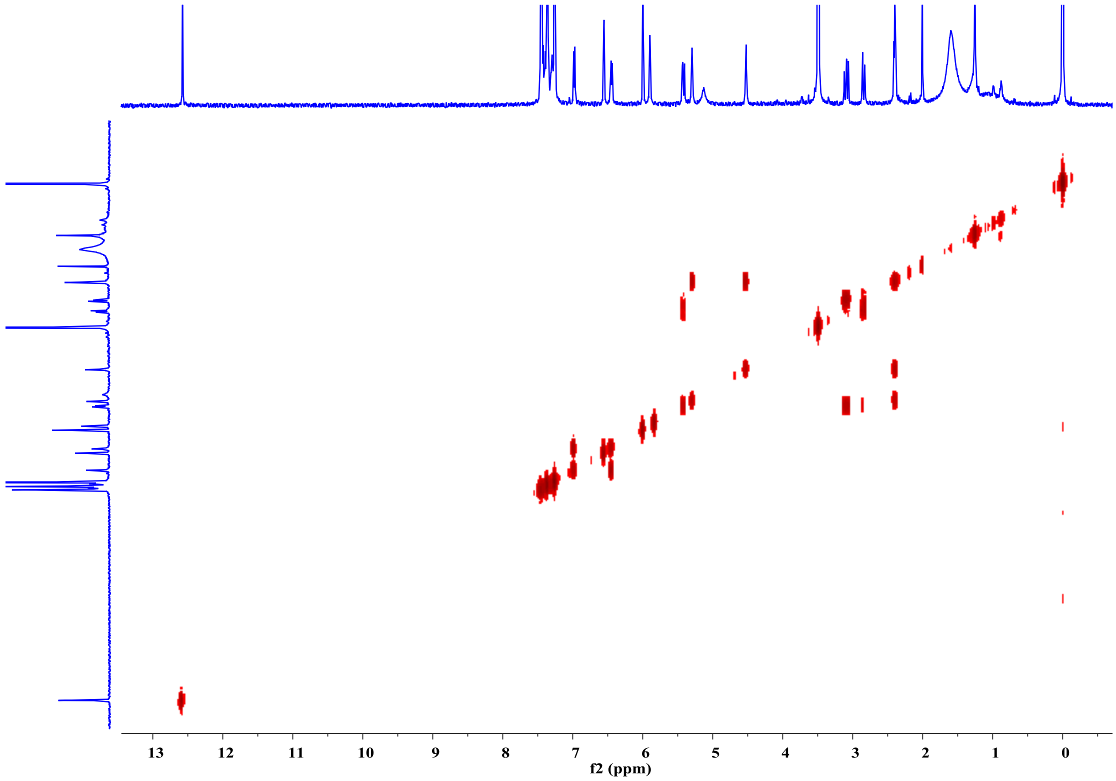

Figure S3. ^{1}H-^{1}H COSY spectrum of oxytrodiflavanone A (**1**) in $CDCl_3$

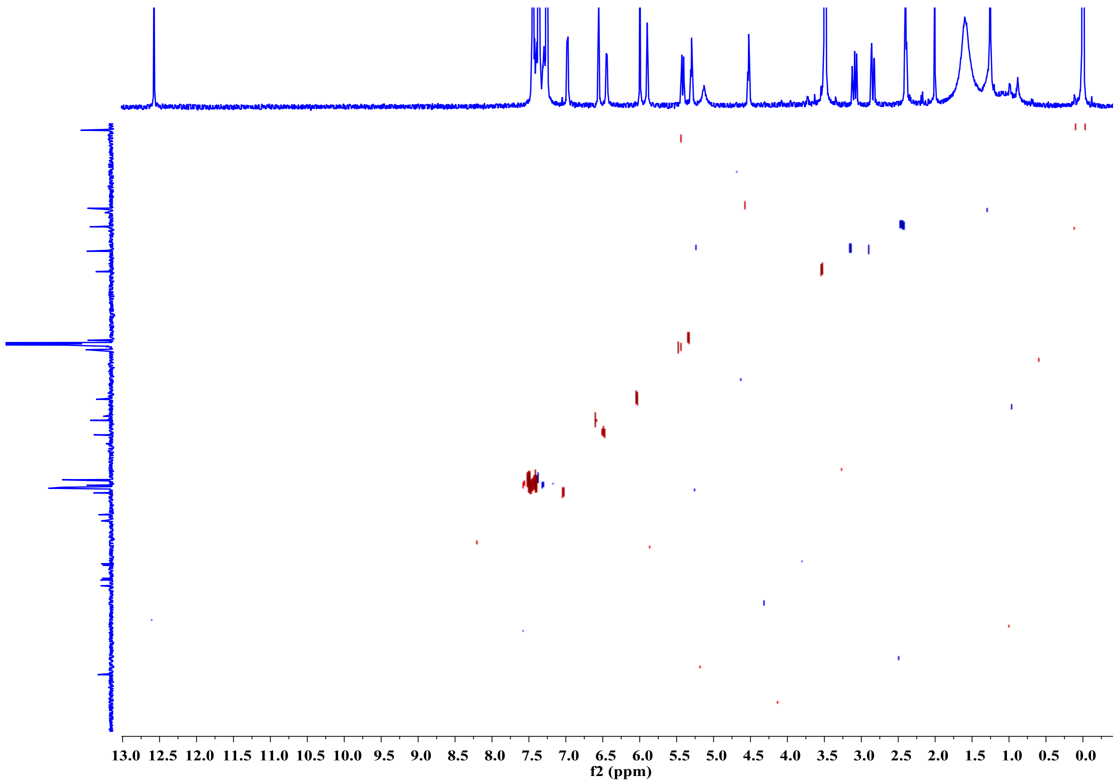

Figure S4. HSQC spectrum of oxytrodiflavanone A (**1**) in $CDCl_3$

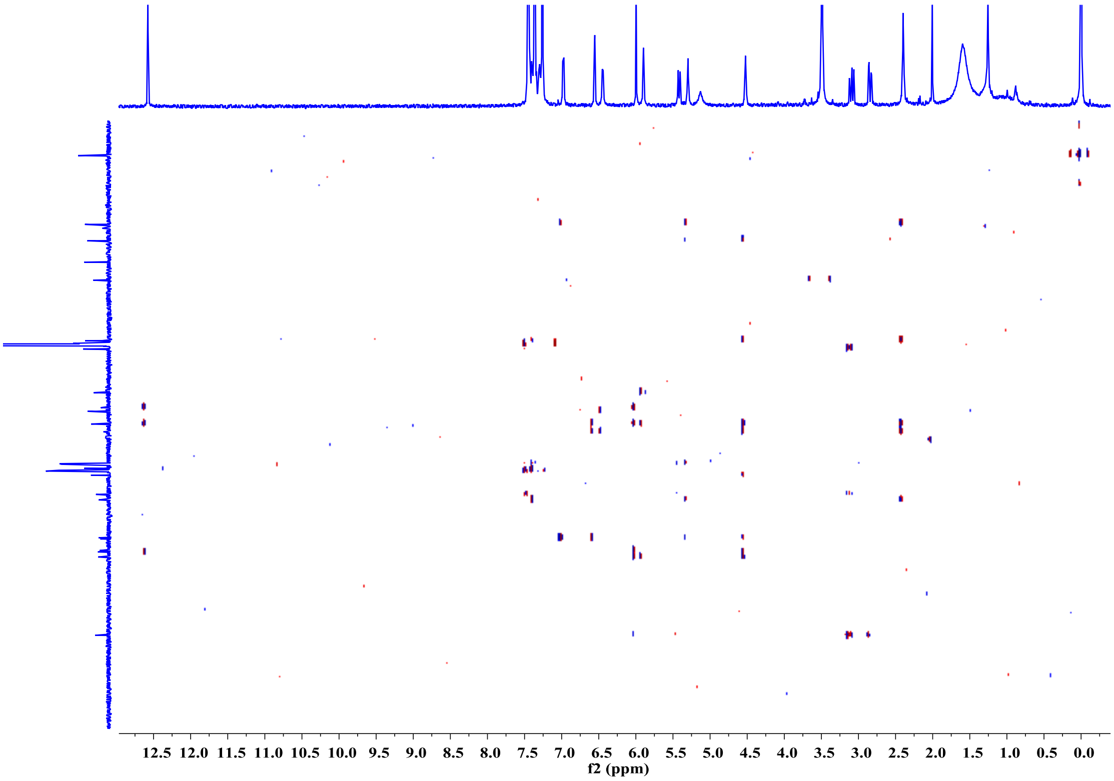

Figure S5. HMBC spectrum of oxytrodiflavanone A (**1**) in $CDCl_3$

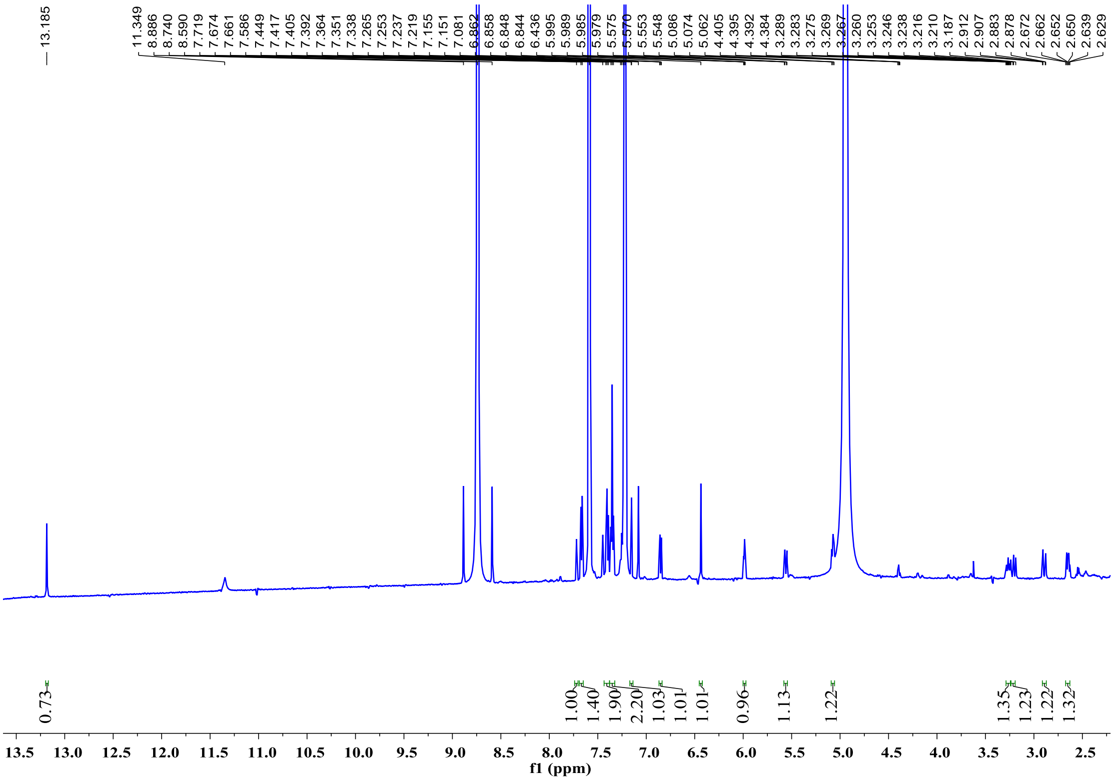

Figure S6. ^{1}H NMR spectrum of oxytrodiflavanone A (**1**) in Pyridine-d_5

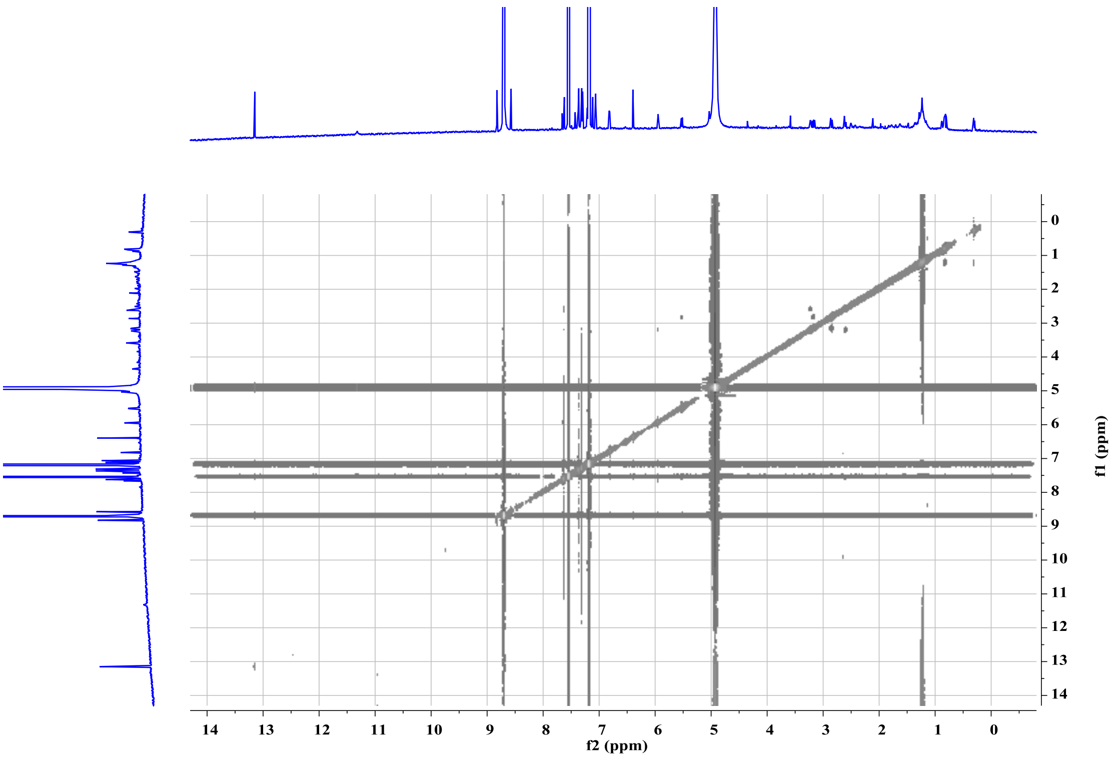

Figure S7. NOESY spectrum of oxytrodiflavanone A (**1**) in Pyridine-d_5

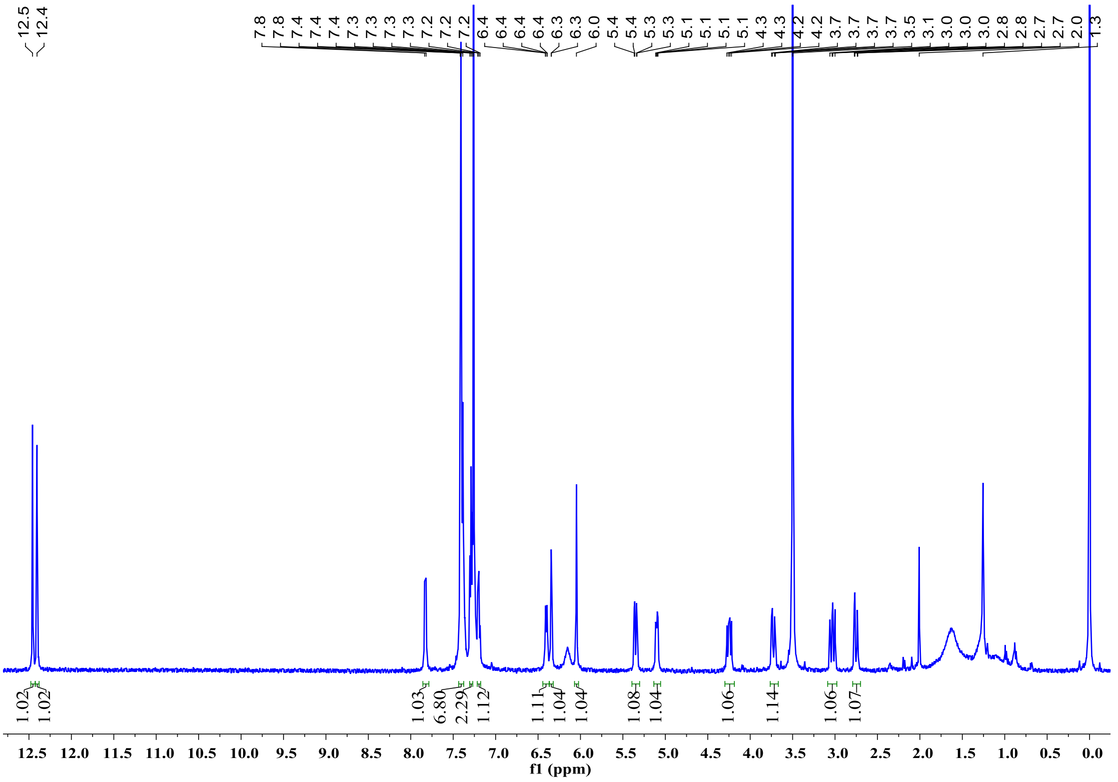

Figure S8. ^{1}H NMR spectrum of oxytrochalcoflavanones A (**2**) and B (**3**) in $CDCl_3$

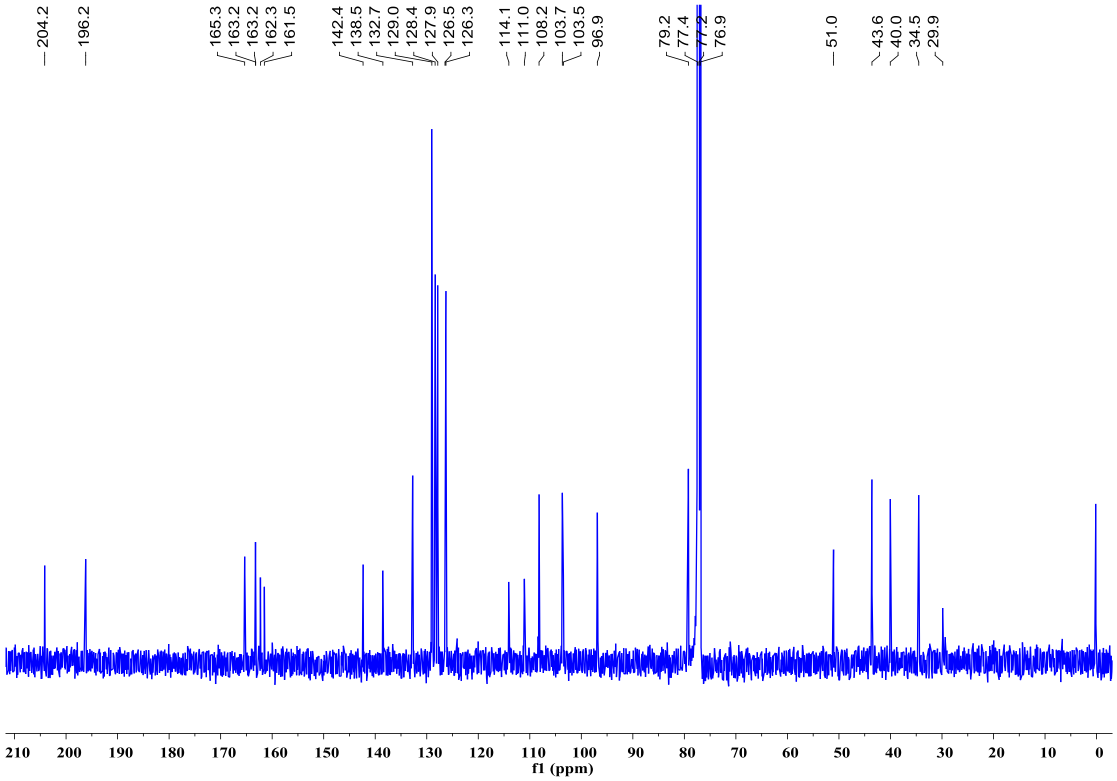

Figure S9. ^{13}C NMR spectrum of oxytrochalcoflavanones A (**2**) and B (**3**) in $CDCl_3$

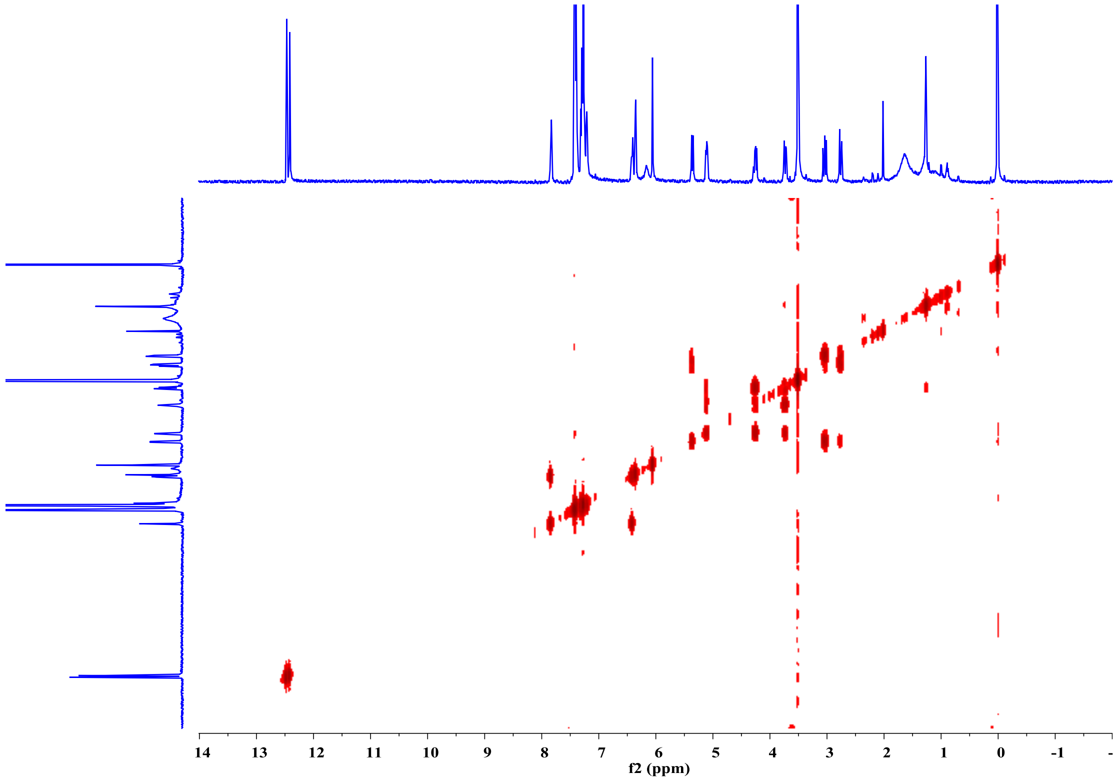

Figure S10. ^{1}H-^{1}H COSY spectrum of oxytrochalcoflavanones A (**2**) and B (**3**) in $CDCl_3$

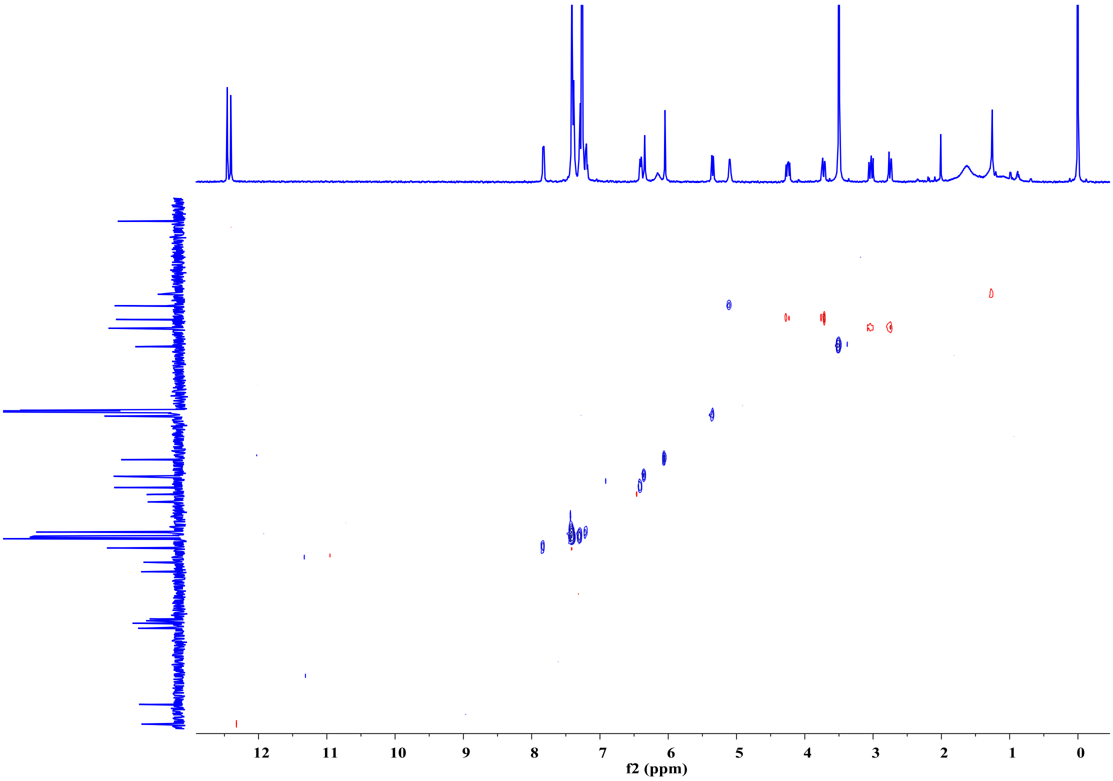

Figure S11. HSQC spectrum of oxytrochalcoflavanones A (**2**) and B (**3**) in $CDCl_3$

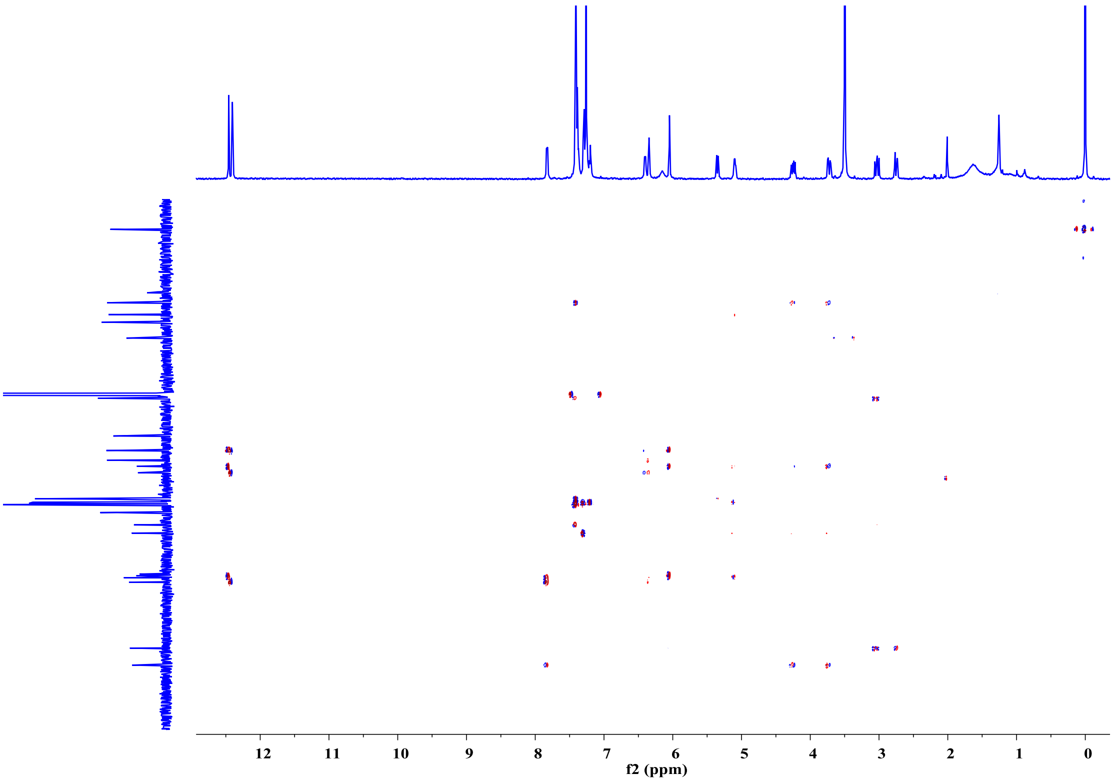

Figure S12. HMBC spectrum of oxytrochalcoflavanones A (**2**) and B (**3**) in $CDCl_3$

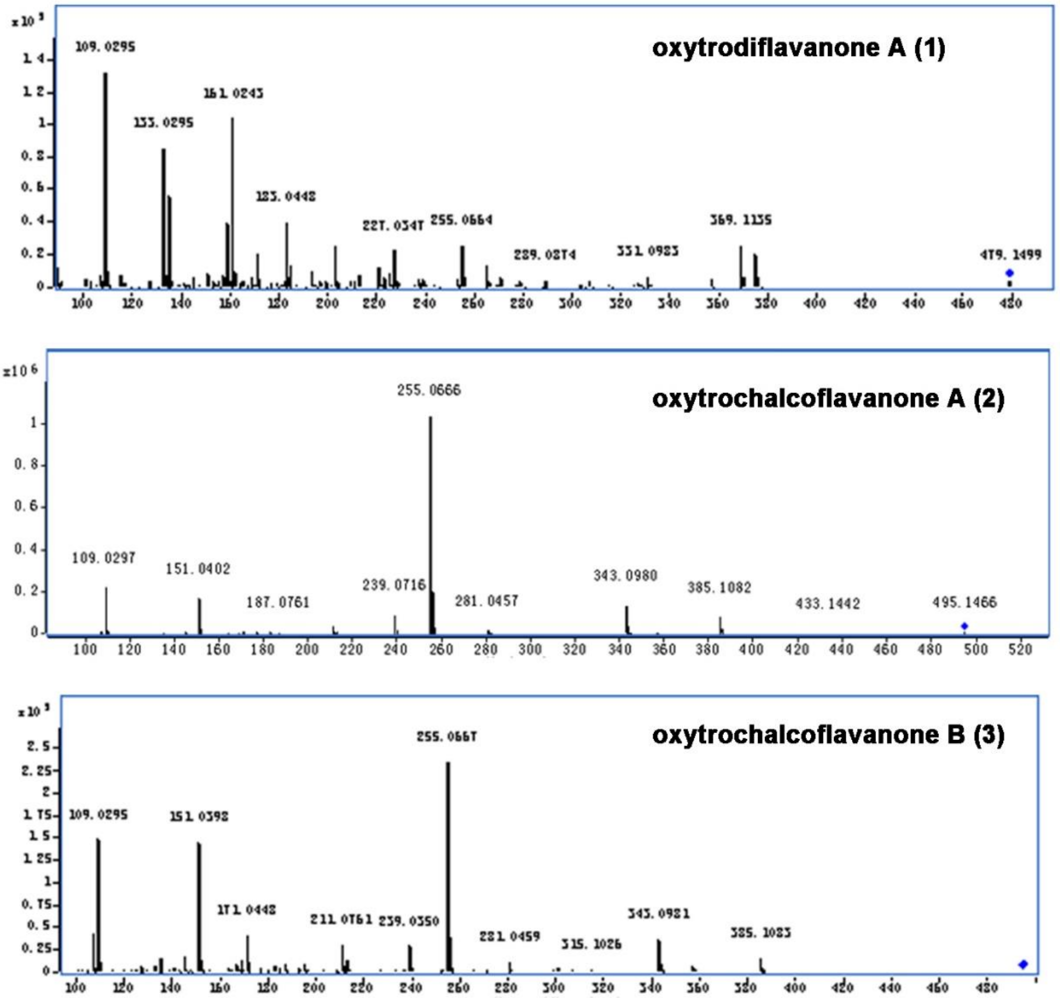

Figure S13. Targeted MS/MS spectra of oxytrodiflavanone A (**1**), oxytrochalcoflavanones A (**2**) and B (**3**) at CE of 25 eV in negative ion mode

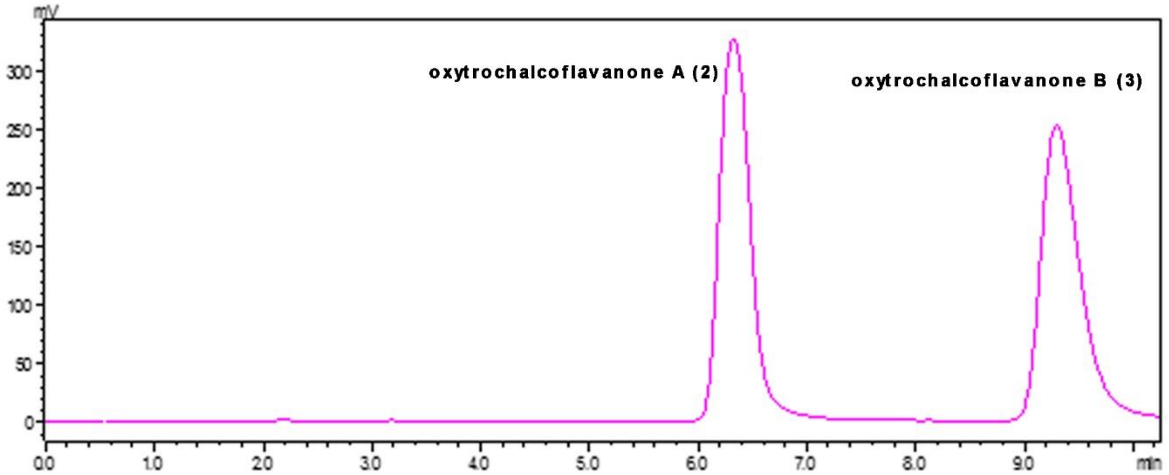

Figure S14. Chiral analysis of oxytrochalcoflavanones A–B (**2-3**)

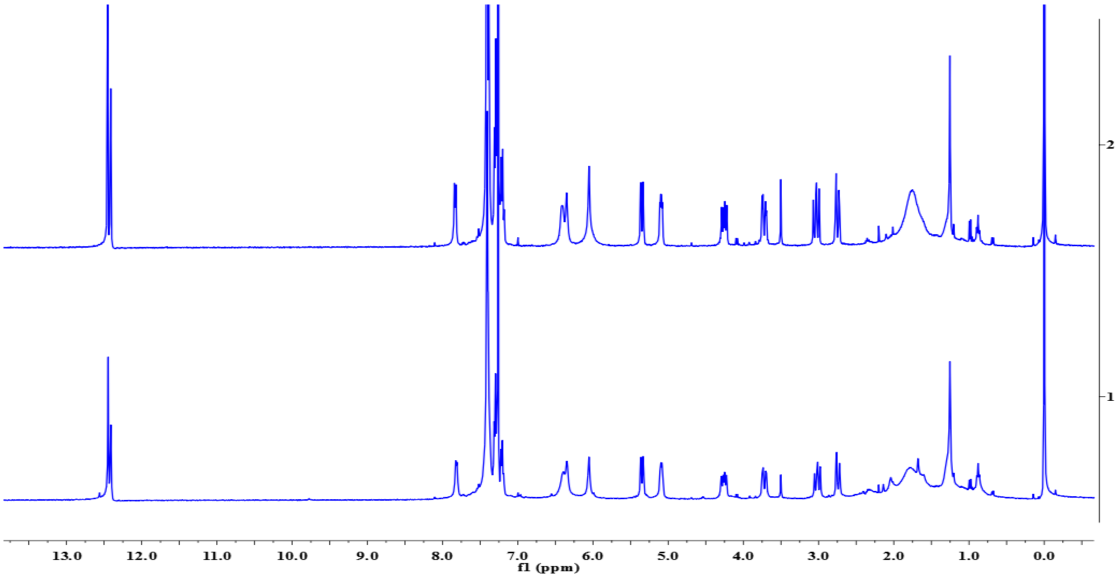

Figure S15.^{1}H NMR spectra of the epimers of oxytrochalcoflavanones A–B (**2-3**)

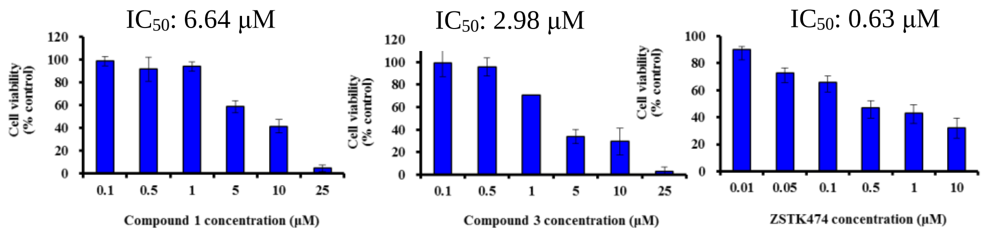

Figure S16. Cell growth inhibitory activities of oxytrodiflavanone A (**1**) and oxytrochalcoflavanone B (**3**) on PC3 cells. The activities of indicated concentrations of Comound 1, Compound 3 and ZSTK474 (postive control) on PC3 cells were determined by MTT assay. Data are presented as mean ± SD, representative of three independent experiments.

Table S1. Raw data of cell growth inhibitory activities of oxytrodiflavanone A (**1**), oxytrochalcoflavanones B (**3**)

Compounds	Concentration (μM)	cell viability (mean±SD, %)
**1**	0.1	98.6 ±4.0
	0.5	91.4 ±10.7
	1	93.8 ±4.1
	5	58.6 ±5.1
	10	41.3 ±5.8
	25	4.9 ±2.9
**3**	0.1	99.4 ±12.4
	0.5	95.5 ±8.1
	1	70.5 ±0.4
	5	33.8 ±6.2
	10	29.4 ±12.1
	25	2.6 ±4.1
ZSTK474	0.01	90.0 ±2.5
	0.05	73.0 ±3.4
	0.1	66.0 ±4.4
	0.5	47.0 ±5.1
	1	43.0 ±6.3
	10	32.0 ±7.5
